# Hibiscus bullseyes reveal mechanisms controlling petal pattern proportions that influence plant-pollinator interactions

**DOI:** 10.1126/sciadv.adp5574

**Published:** 2024-09-13

**Authors:** Lucie Riglet, Argyris Zardilis, Alice L. M. Fairnie, May T. Yeo, Henrik Jönsson, Edwige Moyroud

**Affiliations:** ^1^ The Sainsbury Laboratory, University of Cambridge, 47 Bateman Street, Cambridge CB2 1LR, UK.; ^2^ Department of Genetics, University of Cambridge, Downing Street, Cambridge CB2 3EH, UK.; ^3^ Department of Applied Mathematics and Theoretical Physics, University of Cambridge, Cambridge CB3 0WA, UK.; ^4^ Department of Astronomy and Theoretical Physics, Computational Biology and Biological Physics, Lund University, Lund 223 62, Sweden.

## Abstract

Colorful flower patterns are key signals to attract pollinators. To produce such motifs, plants specify boundaries dividing petals into subdomains where cells develop distinctive pigmentations, shapes, and textures. While some transcription factors and biosynthetic pathways behind these characteristics are well studied, the upstream processes restricting their activities to specific petal regions remain enigmatic. Here, we unveil that the petal surface of *Hibiscus trionum*, an emerging model featuring a bullseye on its corolla, is prepatterned as the bullseye boundary position is specified long before it becomes visible. Using a computational model, we explore how pattern proportions are maintained while petals experience a 100-fold size increase. Exploiting transgenic lines and natural variants, we show that plants can regulate boundary position during the prepatterning phase or modulate growth on either side of this boundary later in development to vary bullseye proportions. Such modifications are functionally relevant, as buff-tailed bumblebees can reliably identify food sources based on bullseye size and prefer certain pattern proportions.

## Teaser

Flowering plants use several mechanisms to control petal pattern dimensions that affect their attractiveness to pollinators.

## INTRODUCTION

The petal epidermis of flowering plants showcases remarkable pattern diversity intricately linked to specialized functions. By combining regions with contrasting features, such as color, texture, or cell morphology, these motifs play a crucial role in pollinator attraction, thus favoring plant reproduction and contributing to speciation (*1*–*4*). Recent studies have uncovered that petal patterns can also fulfill abiotic functions. Ultraviolet (UV)-absorbing flavonoids can modulate transpiration, contributing to heat retention and drought tolerance (*5*–*8*). This could explain why North American populations of common sunflowers (*Helianthus annuus*) found in colder, drier habitats tend to exhibit a larger UV-absorbing bullseye pattern than those growing in warmer and more humid environments (*9*). Similarly, the size of the UV-absorbing center on the corollas of distinct silverweed (*Argentina anserina*) populations correlates with the level of UVB irradiance they experience. Populations closer to the equator often exhibit larger UV-absorbing bullseyes, compared to those found at higher latitudes, possibly providing enhanced protection for pollen grains against UV damage (*10*). These size variations can evolve over extended periods but also in response to environmental fluctuations driven by human activities (*11*). Hence, petal patterns likely represent dual adaptations to both biological and climatic factors, but despite their functional significance, the underlying mechanisms governing their formation remain poorly understood.

How and when spatial patterns of distinct cell types are specified and coordinated as tissues grow, to ultimately give rise to functional organs, remains a fundamental question in developmental biology. Several elegant studies have explored the regulatory processes that govern pigment production across the petal epidermis (*12*–*17*). Those repetitively singled out myeloblastosis (MYB) and basic Helix-Loop-Helix (bHLH) transcription factors (TFs), whose expression patterns account for the accumulation of pigments to specific areas of the petal epidermis. In contrast, our understanding of how distinct cell shapes or cuticle textures are specified across the petal surface remains limited (*18*, *19*). Creating these characteristics seems to rely on precise spatiotemporal control of regulator expression [i.e., the role of the MYB family in epidermal cell differentiation (*20*)] or different biosynthetic pathways [i.e., those involved in the production of distinct cuticular components (*18*)]. While these findings are valuable starting points, the upstream processes that control the restricted expression of genes orchestrating differentiation in neighboring petal cells remain largely unexplored.

Here, we used the flower of *Hibiscus trionum* to explore the onset of pattern formation. Its petals feature a notable bullseye on the adaxial epidermis, with a purple-to-burgundy base contrasting with a white periphery region. Further differences are found at the microscopic level: The proximal epidermal cells, producing dark anthocyanin pigments, are flat, elongated, and covered with a striated cuticle, creating an iridescent blue UV signal visible to pollinators (*21*–*23*). In contrast, the distal cells are white and conical with a smooth cuticle. Both regions are separated by a sharp boundary, invariably located one-third from the petal base in wild-type (WT) individuals. How such a robust boundary is specified and then maintained during petal growth is not yet understood. *H. trionum* belongs to the Trionum complex, a group of hibiscus species broadly distributed across Australasia (*24*). Within this complex, different species and populations exhibit a wide range of bullseye variation (*24*). The mechanisms driving such evolutionary changes in bullseye appearance are unknown and how such diversity impacts pollinator behavior remains to be investigated.

To start addressing these questions, we developed a comprehensive imaging pipeline to capture petal morphogenesis and analyze cell behavior across the entire adaxial epidermis of developing hibiscus petals. We showed that petals are prepatterned as the future domains already exhibit differences in cell expansion and proliferation long before any bullseye feature becomes apparent to the human eye. Using a one-dimensional (1D) computational model, we then identified processes used by plants to maintain or modify bullseye dimensions. Last, we characterized the behavior of buff-tailed bumblebees (*Bombus terrestris*) in response to different bullseye proportions.

## RESULTS

### The adaxial epidermis of *H. trionum* petal is prepatterned

**Fig. 1.**
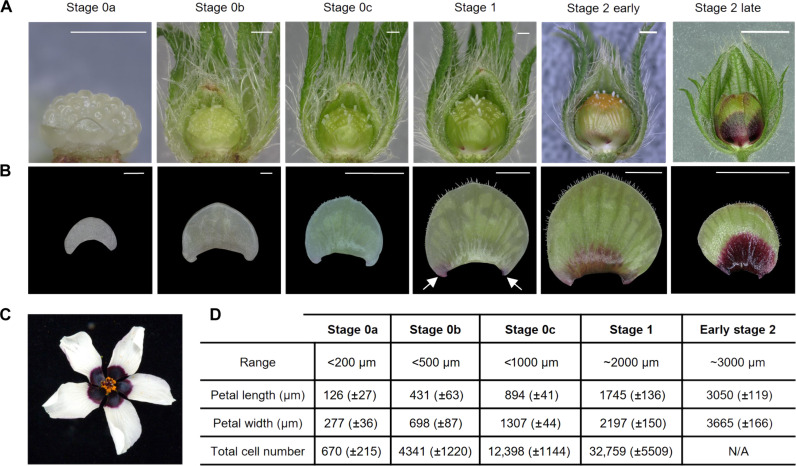
Early development of *Hibiscus trionum* petal. (**A**) Petal organization on flower buds ranging from stage 0a (S0a) to late stage 2 (S2L). The images capture the abaxial side of the petal. Scale bars, 1 mm. (**B**) Early developmental stages of the adaxial petal epidermis, from S0a to late stage 2 (S2L). Pigmentation emerges on both sides of the petal primordium at stage 1 (S1) as indicated by arrows. Scale bars, 100 μm (S0a and S0b), 1 mm (S0c to S2E), and 5 mm (S2L). (**C**) Mature *H. trionum* flower (stage 5). (**D**) Classification criteria for *H. trionum* petal primordia. The total cell count was not assessed at S2E, as only the central petal stripe was imaged at that stage. *n* = 5 petals for each stage.

**Fig. 2.**
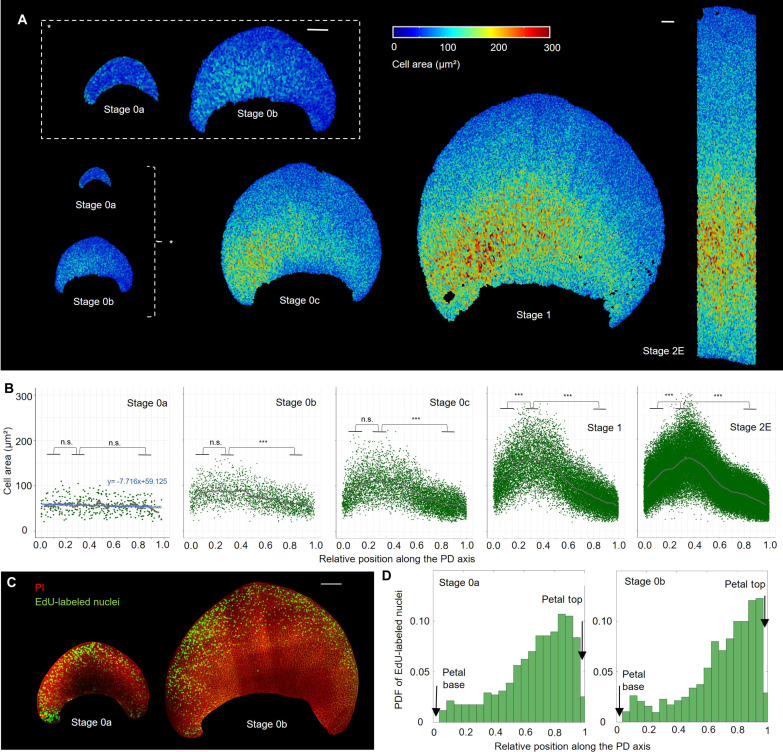
Spatiotemporal distribution of cell expansion and cell division events across the adaxial epidermis during the early stages of *H. trionum* petal morphogenesis. (**A**) Color map of cell area across the WT adaxial petal epidermis during early developmental stages (from S0a to S2E). Scale bars, 100 μm. (**B**) Cell area distribution across the PD axis of the *H. trionum* petal. The graphs consider only the central stripe of cells (20% of the petal width) for readability. Cell positions along the PD axis are relative (0 = petal base; 1 = petal tip). Gray lines correspond to the rolling average of cell area of all replicates. Statistical differences were calculated using a Shapiro-Wilk test to evaluate the normality and then a *t* test; n.s., nonsignificant, ***P < 0.01. *n* = 5 petals for each stage. (**C**) Distribution of cell division events across the adaxial epidermis of S0a and S0b petals. Newly synthesized DNA is labeled using fluorescently labeled nucleotide analog 5-ethynyl-2-deoxyuridine (EdU; green) and plasma membranes are stained with PI (red). Scale bar, 100 μm. (**D**) Probability density function (PDF) of the EdU-labeled nuclei along the PD axis of *H. trionum* S0a petals (left) and S0b (right) (stripes corresponding to 20% of the petal width and centered along the PD axis were analyzed, see fig. S1E). *n* = 5 petals for each stage.

To start understanding how robust bullseyes form on the petal surface of *H. trionum,* we imaged the adaxial epidermis during successive floral developmental stages (*25*). The dark pigmentation is the first visible element of the pattern to emerge (Fig. 1, A and B). At stage 0 (S0), the petal surface is entirely green, showing no evident sign of cellular differentiation (Fig. 1, A and B). The appearance of color on both sides of the petal attachment point to the base of the floral structure is characteristic of stage 1 (S1) (Fig. 1, A and B). By the end of early stage 2 (S2E), the basal region of the primordium is fully pigmented, with a sharp boundary demarcating the proximal and distal domains (*25*) (Fig. 1, A and B). To identify when and how this bullseye boundary emerges, we gathered quantitative cellular growth data across the petal epidermis at high spatiotemporal resolution. Until the flower opens (Fig. 1C), petals are enclosed within the bud, with their adaxial epidermis facing inward, posing technical challenges for observation (Fig. 1A). To generate a reference dataset describing epidermal cell behavior at cellular resolution, we used confocal microscopy and captured images of dissected petals stained with FM1-43 that labels cell outlines. Following image segmentation with MorphoGraphX (*26*), we quantified global changes in cell number and primordia morphology at fixed time points of petal development (Figs. 1D and 2A). This analysis led us to subdivide the S0 phase into three sub-stages: stage 0a (S0a), 0b (S0b), and 0c (S0c) (Fig. 2A).

We found cell area to be uniform across the adaxial petal epidermis at S0a (Fig. 2A), but by S0b, heterogeneity emerges as larger cells appear on one side of the petal. By S0c, the zone with larger cells expands from one petal side in a croissant-shaped pattern, resulting in right-left petal asymmetry (Fig. 2A). This motif becomes more pronounced toward S1 and S2E. We quantified the distribution of cell area along the proximo-distal (PD) axis, focusing on a central epidermal stripe (20% of the petal width), and confirmed that cell area is uniform at S0a (Fig. 2B, fig. S1A, Materials and Methods, and Table 1 for statistics). From S0b, cell area is distributed heterogeneously, with larger cells preferentially located in the basal section of the adaxial petal epidermis (Fig. 2B, Materials and Methods, and Table 1). From S0c onward, maximum cell area peaks around one-third of the petal length from the base (Fig. 2B, Materials and Methods, and Table 1). This peak of larger cells along the PD axis sharpens at S1, and the relative position of this maximum is maintained as petal primordia grows to reach S2E (Fig. 2B, Materials and Methods, and Table 1). In addition, from S1, cells in the proximal part of the petal epidermis begin to elongate, exhibiting a significantly higher aspect ratio than those in the distal region (fig. S1, B and C). Conversely, distal cells that will become conical by S4 (*25*) already display a higher circularity than proximal cells around the one-third position at S1, although circularity is highly variable across the epidermis at such an early stage (fig. S1D). Thus, cell area and geometry are distinctly regulated along the PD axis of the petal, with differences emerging in a croissant-shaped pattern during the S0 phase, long before mature bullseye features (pigmentation, cuticular ridges, and contrasting cell shapes) appear. Notably, this early pattern is characterized by a landmark position at one-third of the petal length, where the largest cells are located. This position coincides with the position of the future bullseye boundary (figs. S2, A to E, and S3 to S5).

**Table 1.** *Hibiscus trionum*, cell area distribution by stages.

Stages	Section along the PD axis	Mean area (μm^2^)	Test comparing mean area in:	Results
Stage 0a	[0.05;0.15[	57.78	[0.05;0.15[ and [0.25;0.35[	*t* = 0.23452, df = 8, *P* = 0.8205
	[0.25;0.35[	56.35	–	–
	[0.8;0.9[	53.2	[0.8;0.9[ and [0.25;0.35[	*t* = 0.65003, df = 8, *P* = 0.5339
Stage 0b	[0.05;0.15[	83.43	[0.05;0.15[ and [0.25;0.35[	*t* = −0.59716, df = 8, *P* = 0.5669
	[0.25;0.35[	86.8	–	–
	[0.8;0.9[	59.45	[0.8;0.9[ and [0.25;0.35[	*t* = 6.5047, df = 8, *P* = 0.0001872
Stage 0c	[0.05;0.15[	94.1	[0.05;0.15[ and [0.25;0.35[	*t* = −2.0172, df = 8, *P* = 0.0784
	[0.25;0.35[	111.2	–	–
	[0.8;0.9[	60.52	[0.8;0.9[ and [0.25;0.35[	*t* = 9.6018, df = 8, *P* = 1.149 × 10^−5^
Stage 1	[0.05;0.15[	94.1	[0.05;0.15[ and [0.25;0.35[	*t* = −6.9427, df = 8, *P* = 0.0001193
	[0.25;0.35[	111.2	–	–
	[0.8;0.9[	60.52	[0.8;0.9[ and [0.25;0.35[	*t* = 18.208, df = 8, *P* = 8.508 × 10^−8^
Stage 2E	[0.05;0.15[	94.1	[0.05;0.15[ and [0.25;0.35[	*t* = −9.9462, df = 8, *P* = 8.837 × 10^−6^
	[0.25;0.35[	111.2	–	
	[0.8;0.9[	60.52	[0.8;0.9[ and [0.25;0.35[	*t* = 24.453, df = 8, *P* = 8.352 × 10^−9^

Next, we examined cell proliferation during the prepatterning phase by mapping cell division events using the fluorescent nucleotide analog, 5-ethynyl-2-deoxyuridine (EdU) (*27*), which labels newly replicated DNA. We found that cell proliferation events are not uniformly distributed across the S0a petal epidermis, as most EdU-labeled nuclei resided in the distal region (Fig. 2C), mirroring the forthcoming bullseye layout. Quantifying the distribution of EdU-labeled nuclei along the petal PD axis (Fig. 2D and fig. S1E) confirmed that division events are mainly restricted to the upper half of the epidermis at S0a, with the highest proportion of fluorescent nuclei near the top of the petal (Fig. 2D). This distribution persists throughout the prepatterning phase to S1 (Fig. 2, C and D, and fig. S1, F and G). Thus, cell proliferation is differentially regulated across the two main regions of the bullseye (distal versus proximal) very early during petal development.

Together, our results suggest that cell properties across the adaxial epidermis of the hibiscus petal are prepatterned long before the distinctive features of the bullseye (pigmentation, cuticular ridges, and contrasting cell shapes) become visible. This early pattern is characterized by differences in cell division between the proximal and distal regions and a croissant-shaped distribution of cell size, with the largest cells lying at the one-third mark along the petal PD axis, potentially specifying the position of the final bullseye boundary.

### The early pattern boundary coincides with the mature bullseye boundary

**Fig. 3.**
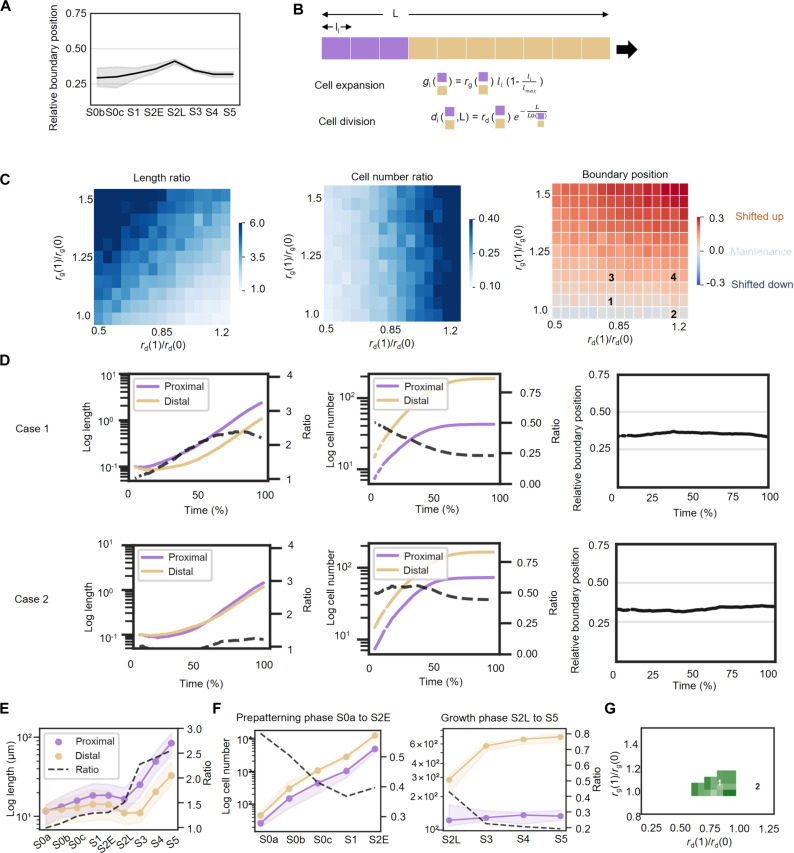
Principles governing the maintenance of the relative boundary position along the PD axis during *H. trionum* petal development. (**A**) Evolution of the boundary position during WT *H. trionum* petal development. The boundary position is automatically detected as the peak of largest cells in window-averaged cell area for S0a to S2E (prepattern boundary) and corresponds to the pigmentation boundary, when pigmentation shifts from pink to white (bullseye boundary) for S2L to S5. (**B**) Investigation of boundary maintenance principles through a 1D cell model featuring two distinct cell fates (representing proximal and distal epidermis cells—see Materials and Methods). (**C**) Analysis of three key observables from the simulation of the developmental process with varying ratios of growth parameters (expansion and division rates) for the two cell types: average-length ratio and number-of-cell ratio between the two cell types, and (right) deviation of boundary position from the initial one-third position (see Materials and Methods for details on calculation). (**D**) Detailed plots of simulations where the model predicts boundary maintenance. (**E**) Evolution of the median adaxial epidermis cell length in the proximal (below boundary) and distal (above boundary) regions during WT *H. trionum* petal development. The ratio represents the length of the proximal cells divided by that of the distal ones. (**F**) Evolution of the average cell numbers in the proximal and distal regions during early stages (left, stage 0a to stage 2E) and late stages (right, stage 2L to stage 5) of WT *H. trionum* petal development. (**G**) An objective function representing the average percentage distance over time between simulated and experimental values for the three observables from (C). The green region indicates conditions in parameter space where this distance is less than 20% (see Materials and Methods definition of the objective function). The color shade reflects the distance, with lighter shades indicating simulations closer to experimental observations and darker shades representing greater deviations.

To understand the relationship between early patterning and bullseye formation, we analyzed the distribution of cell features along the PD axis from late stage 2 (S2L) to stage 5 (S5, maturity) in *H. trionum* WT petals (fig. S2, A to E). First, we tracked the position of the pigmentation boundary, which marks the transition from pink to white (fig. S2E). The large size of these petals makes them unsuitable for cellular resolution imaging and segmentation with our pipeline. Consequently, we manually measured cell features (area, aspect ratio, and circularity) along a single row of cells from the base to the top of the petal adjacent to the mid-vein and found that the pigmentation transition consistently aligns with changes in cell area (fig. S2E). We plotted the evolution of the boundary position (early pattern boundary from S0b to S2E and bullseye boundary from S2L to S5) across developmental stages in *H. trionum*. After its initial establishment one-third from the petal base during the early patterning phase, the relative position of the boundary transiently rises to reach 0.4 at S2L before stabilizing back around the one-third mark at S4 (Fig. 3A). Although the proximal and distal regions exhibit different growth properties, bullseye proportions remain mostly constant throughout petal morphogenesis in terms of cell division and expansion. The growth disparities between these regions must thus be reconciled to maintain the relative lengths of the two zones that make the bullseye motif.

### Principles governing boundary maintenance throughout petal growth

To mechanistically understand how bullseye proportions are conserved despite the significant growth that the petal primordium experiences to reach maturity, we developed a computational model of growth. We focused on how the interplay between differential growth and cell division affects boundary position. Specifically, we aimed at identifying conditions supporting the maintenance of the bullseye proportion established during the prepatterning phase. Our experimental analysis focused on the relative position of the boundary along the PD axis, leading us to model space as a 1D linear array of cells. This spatial simplification has been used in other studies where one dimension of the tissue dominates, for example when considering hormone distribution in the root (*28*). The initial state consists of a single line of 21 cells (Fig. 3B). The model incorporates the following assumptions: two cell types representing proximal and distal cells, with growth and division rates of each cell type depending on their respective fates (Fig. 3B). Initially, the ratios of these cell types are set at the one-third position from the base, and all cells are of uniform length. This assumes that while cell morphology is identical, proximal or distal cell fates have already been specified. According to our data (Fig. 2, A and B), this occurs between stage 0a and stage 0b. Then, we simulated petal development by applying growth and division rates to individual cells while also decreasing cell division rates over time and slowing cell expansion rates as cells reached their maximum size (Fig. 3B; fig. S3, A and B; and Materials and Methods).

To identify conditions that led to pattern proportion maintenance, we explored the change in relative boundary position for a range of expansion and division rates between the two petal regions expressed as ratios (e.g., an expansion ratio of 2 indicates that proximal cells expand twice as fast as distal cells, while an expansion ratio of 1 corresponds to an expansion rate equal in both regions). We identified a region in parameter space that leads to conservation of bullseye proportions (Fig. 3C). In this region, we observed an anti-correlation between growth and division ratios, with one compensating for the other, e.g., an increase in proximal growth is counterbalanced by a proportional decrease in division rate, thereby preserving the relative size of the two regions and consequently the relative position of the boundary. The impact of other parameters on the pattern proportions appears relatively minor (fig. S8).

To explore differences between scenarios, we also plotted outcomes of the model in terms of ratio of lengths and number of cells between two regions (as suggested by the observed differences in cell area and cell number between the two regions, Fig. 2) and elaborated on two representative cases that mirror each other on either side of the (1, 1) point (equal growth and division) in the explored ratio space (Fig. 3, C and D). In those configurations, maintenance of bullseye proportions is achieved either when the expansion rate is higher in the proximal domain, with a higher cell division rate in the distal region (case 1, Fig. 3, C and D), or when the division rate is higher in the proximal domain but with similar growth rate in both zones (case 2, Fig. 3, C and D).

To test whether one of these two scenarios matches the pattern of cell behavior and the boundary maintenance observed in *H. trionum* (Fig. 3A), we experimentally characterized parameters of expansion and division at later stages of development. Given the technical challenges of directly tracking petal cell growth parameters over time, we adopted an approach that leverages averaged behaviors across developmental stages to reveal fundamental trends. We plotted the average cell length in both proximal and distal regions across stages (Fig. 3E) to deduce effective cell expansion rates (product of both division and expansion), and similarly, we quantified cell number in each region to approximate the division rates (Fig. 3F). Overall, the development of *H. trionum* petal occurs in two phases: an initial phase marked by intensive cell division activity, followed by a subsequent phase characterized by pronounced cell expansion (Fig. 3, E and F). Notably, division events occur more frequently at early stages (Fig. 3F and fig. S2F), and the division phase lasts longer in the distal region than in the proximal domain. The division phase continues until S3 in the distal part of the bullseye but stops earlier, around S2E-S2L, in the proximal domain (Fig. 3F and fig. S2F). As a result, from S3 onward, the number of distal cells along the PD axis is five times higher than the number of proximal cells (Fig. 3F). Those proximal cells exhibit higher effective growth rates (Fig. 3E and fig. S2F). We found that from S3 onward, proximal cells are approximately twice as long as the distal cells despite cell dimensions being relatively even within the two domains at the start of petal development (Figs. 3E and 2B). Despite such growth disparities, the boundary position remains mostly constant throughout petal development, except for the distinct “bump” at S2L (Fig. 3A). This bump can be attributed to the asynchrony in the exit from the division phase between the two regions (Fig. 3F).

In summary, we found that in WT *H. trionum*, the expansion rate is higher in the proximal domain and division events occur more frequently in the distal region. These experimental conditions (length ratios and number-of-cell ratios) do not match with the second scenario in the model (case 2, Fig. 3G) where a higher division rate ratio combined with similar growth rates in the two zones maintains the boundary position but leads to cell sizes and number-of-cell ratios that differ from those experimentally recorded in WT *H. trionum* (case 2, Fig. 3, D to G). However, they align with the outcomes theoretically predicted by the first scenario of our simulations (case 1, Fig. 3, D to G). Altogether, we identified that the boundary is set very early during the first stages of petal development and that local differences in cell expansion and division between the two early domains enable its maintenance, ultimately yielding the mature bullseye pattern.

### Developmental processes responsible for changes in bullseye proportions

**Fig. 4.**
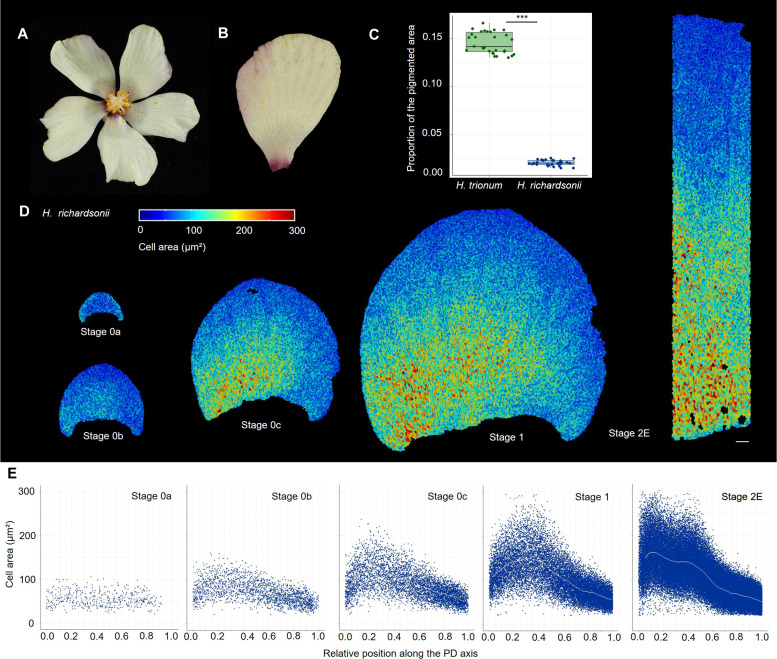
The prepattern boundary is specified closer to the petal base during early stages of *H. richardsonii* petal development. (**A**) Flowers of *H. richardsonii* display a smaller bullseye than its sister species *H. trionum*. (**B**) Close-up view of *H. richardsonii* petals. (**C**) Comparison of bullseye proportions (pigmented area/total area) in *H. trionum* and *H. richardsonii* open flowers (stage 5). *n* = 10 flowers and 3 petals per genotype. Statistical differences were calculated using a Shapiro-Wilk test to evaluate the normality, followed by *t* test, ***P < 0.01. (**D**) Color map of cell area across the adaxial petal epidermis of *H. richardsonii* during early developmental stages (from S0a to S2E). Scale bar, 100 μm. (**E**) Cell area distribution across the PD axis of *H. richardsonii* petals. The graph considers only the central stripe of cells (20% of the petal width) for readability. Cell positions along the PD axis are relative, with 0 corresponding to the petal base, and 1 to the petal tip. Gray lines correspond to the average cell area of all replicates. *n* = 5 petals for each stage.

To start examining the processes plants use to regulate bullseye size, we took advantage of the natural diversity within the Hibiscus family and characterized pattern formation in *Hibiscus richardsonii*, a close relative of *H. trionum* that produces flowers with notably smaller bullseyes (Fig. 4, A and B). In *H. richardsonii,* the pigmented area represents only 2.1% of the total petal surface, a notable contrast to the 14.5% observed in *H. trionum* (Fig. 4C).

In addition to the shift in pigmentation, the bullseye is also smaller in terms of cell shape distribution. When flowers open (S5), the cell shape boundary (transition from flat striated tabular to conical smooth cells) is closer to the petal base, with the maximum cell size lying at the 0.15 position along the PD axis of the petal (fig. S5A). Beyond this peak, cell area declines to reach a plateau at S2E around the 0.3 to 0.4 position before decreasing again sharply. To investigate the mechanism responsible for reduced bullseye size in this closely-related Hibiscus species, we tracked early pattern boundary formation. From S0a to S0b, we observed the same tendency in cell area distribution between *H. richardsonii* and *H. trionum* petal primordia (fig. S5B). However, at S0c, while larger cells emerge around the one-third position from the base in *H. trionum* (Fig. 2, A and B), larger cells are found closer to the petal base in *H. richardsonii* (near the 0.2 position from the base) (Fig. 4, D and E). By S2E, instead of occupying the one-third position, the largest cells are found nested closer to the base (averaged cell area maxima observed at 0.15 position from the base) and, on average, are smaller than their equivalent at the one-third position in *H. trionum* (around 140 μm^2^ for *H. richardsonii* versus 165 μm^2^ for *H. trionum*) (fig. S5B). Cell size then drops significantly in the distal half of the petal, following a trend already observed in *H. trionum* primordia. Thus, the reduction in bullseye dimensions that occurred on the lineage leading to *H. richardsonii* is associated with a change in cell behavior along the petal PD axis, with the early pattern boundary specified closer to the petal base. Together our results suggest that the size reduction of the structural bullseye (cell shape and texture) in *H. richardsonii* is due to changes affecting the prepatterning phase at S0, with an early boundary positioned closer to the petal base and maintained at that position during the later growth phase, as observed in *H. trionum*.

**Fig. 5.**
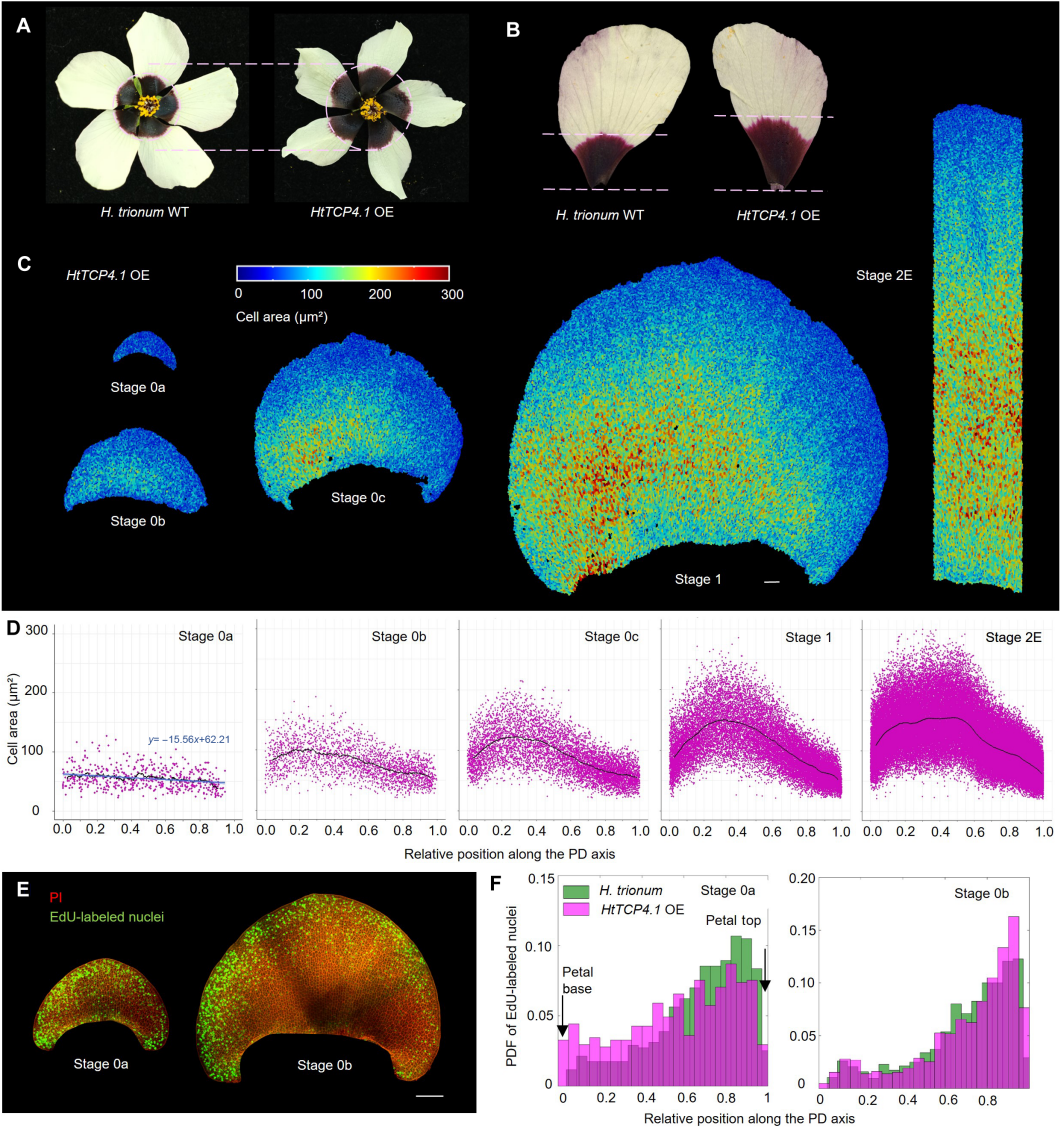
Overexpression of *HtTCP4.1* produces *H. trionum* flowers with a larger bullseye due to a change in spatial distribution of cell division events across the adaxial petal epidermis. (**A**) Flowers of *HtTCP4.1* overexpression (OE) transgenic lines display a larger bullseye compared to WT. (**B**) Close-up view of WT (left) and *HtTCP4.1* OE (right) petals. The pink dotted lines indicate the distance between the petal base and the bullseye boundary. (**C**) Color map of cell area across the adaxial epidermis of *HtTCP4.1* OE petals during early developmental stages (from S0a to S2E). Scale bar, 100 μm. (**D**) Cell area distribution across the PD axis of *HtTCP4.1* OE petals. The graphs consider only the central stripe of cells (20% of the petal width) for readability. Cell positions along the PD axis are relative, with 0 corresponding to the petal base, and 1 to the petal tip. Black lines correspond to the average cell area of all replicates. *n* = 5 petals for each stage. (**E**) Distribution of cell division events across the adaxial epidermis of S0a and S0b *HtTCP4.1* OE petals. Newly synthesized DNA is labeled using fluorescently labeled nucleotide analog 5-ethynyl-2-deoxyuridine (EdU; green) and plasma membranes are stained with PI (red). Scale bar, 100 μm. (**F**) Probability density function (PDF) of the EdU-labeled nuclei along the PD axis of *HtTCP4.1* OE S0a and S0b petals compared to *H. trionum* WT (stripes corresponding to 20% of the petal width and centered along the PD axis were analyzed, see fig. S6E). *n* = 5 petals for each stage. A chi-square test for independence was used to compare the distribution of cell division event along the PD axis for *H. trionum* versus HtTCP4.1, and demonstrated that it was significantly different for stage 0a (χ^2^ = 51.09, *P* < 0.001) but not for stage 0b (χ^2^ = 13.03, *P* = 0.16).

Theoretically, a second mechanism, acting later during petal development, could account for changes in bullseye proportions: The early boundary could remain specified at the one-third position (prepatterning phase unchanged), but cell expansion and proliferation could vary on either side of this boundary during the growth phase, yielding a shifted bullseye boundary in mature flowers. To test this hypothesis, we therefore turned our attention toward candidate genes likely to control cell growth and proliferation in developing petals. TFs from the TEOSINTE BRANCHED 1, CYCLOIDEA, PCF1 (TCP) family are plant-specific transcriptional regulators (*29*–*32*) that play a pivotal role in various developmental processes primarily by controlling cell growth, proliferation, and differentiation (*33*). In WT *H. trionum*, *HtTCP4.1* and its paralog *HtTCP4.2* are both preferentially expressed in the distal petal region throughout petal development (fig. S6C). We found that when placed under control of the strong constitutive 35S promoter, the ectopic activity of *HtTCP4.1* produced flowers with increased bullseye proportions as the pigmented area represented 25% of the total petal area instead of 14.5% recorded in WT flowers (Fig. 5, A and B, and fig. S6A). A similar phenotype was obtained when we constitutively overexpressed *HtTCP4.2* (see fig. S6, A and B).

We observed a similar cell area distribution along the PD axis during the prepatterning phase (from S0a to S1) in *HtTCP4.1* overexpression (OE) petals compared to WT (Fig. 5, C and D, cf. Fig. 2, A and B): Cell area is on average uniform across the epidermis at S0a, and an early croissant-shaped pattern emerges at S0b, resulting in a right-left asymmetry (Fig. 5C). At S0c, cell area peaks at one-third of the petal length from the base, as in WT (Fig. 5D). By S1, the maximum cell size at the peak position is similar for both *HtTCP4.1* OE and WT (*t* = 1.38, *P* > 0.05) (Figs. 2B and 5D, Materials and Methods, and Table 1). This suggests that the mechanism leading to a larger bullseye in transgenic individuals constitutively overexpressing *HtTCP4.1* is not a change in the specification of the early boundary during the prepatterning phase. At S2E, we observed a higher proportion of large cells in the proximal region of the petal in *HtTCP4.1* OE petals compared to WT (Fig. 5C). Those petals display a plateau of larger cells starting closer to the petal base and expanding beyond the one-third landmark of the petal length (Fig. 5D), a trend that can also be observed later along the PD axis of the mature petal at S5 (fig. S7C). While both WT and transgenic petals reach the same maximum average cell size at S2E (*W* = 46, *P* > 0.05) (Figs. 2B and 5D), the proportion of cells reaching this size is significantly increased when *HtTCP4.1* is overexpressed (*W* = 0, *P* < 0.005). Cell division events follow similar distributions in *HtTCP4.1* OE and WT petal primordia from S0b to S1 (χ^2^ = 13.03, *P* = 0.16) (Fig. 5, D and F, and fig. S6, F and G). However, at S0a, the distributions of the cell division events are significantly different between the two genotypes (χ^2^ = 51.09, *P* < 0.001). The active proliferation zone is not restricted to the distal part of the petal, as observed in WT, but EdU-labeled nuclei are detected across the entire adaxial epidermis of *HtTCP4.1* OE instead, including the proximal region (Fig. 5, E and F, and fig. S6E), indicating that cell proliferation is more uniform across the adaxial petal epidermis when *HtTCP4.1* is constitutively overexpressed.

Altogether, these findings indicate that the larger bullseye in *HtTCP4.1* OE is not due to a shift in early boundary positioning along the petal PD axis during the prepatterning phase but might instead be due to an increase in cell proliferation at the petal base. These additional basal cells, assigned to the proximal domain, are programmed to grow more than the distal ones, and this could explain the higher proportion of larger cells at the petal base, ultimately resulting in a larger bullseye.

**Fig. 6.**
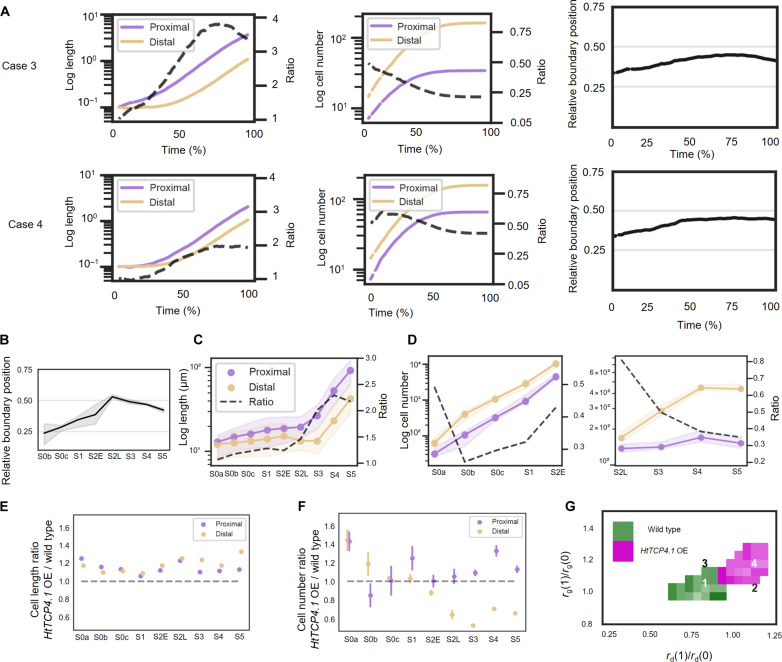
Evolution of the boundary position and epidermis cell features during petal development in transgenic lines overexpressing *HtTCP4.1*. (**A**) Detailed plots of simulations where the model predicts a shift in the boundary position. (**B**) Evolution of the boundary position during *HtTCP4.1* OE petal development. The boundary position is automatically detected as the peak of largest cells in window-averaged cell area for S0a to S2E (prepattern boundary) and corresponds to the pigmentation boundary, when pigmentation shifts from pink to white (bullseye boundary) for S2L to S5. (**C**) Evolution of the median adaxial epidermis cell length in the proximal (below boundary) and distal (above boundary) regions during *HtTCP4.1* OE petal development. The ratio represents the length of the proximal cells divided by that of the distal ones. (**D**) Evolution of the average cell numbers in the proximal and distal regions during early stages (left, stage 0a to stage 2E) and late stages (right, stage 2L to stage 5) of *HtTCP4.1* OE petal development. (**E**) Comparison of median cell length in *HtTCP4.1* OE versus WT in the proximal and distal regions during *H. trionum* petal development. (**F**) Comparison of average cell numbers in *HtTCP4.1* OE versus WT in the proximal and distal regions during *H. trionum* petal development. (**G**) An objective function representing the average percentage distance over time between simulated and experimental values for the three observables. Colored regions (green for WT and purple for *HtTCP4.1* OE) indicate conditions in parameter space where this distance is less than 20% (see Materials and Methods definition of the objective function). The color shade reflects the distance, with lighter shades indicating simulations closer to experimental observations and darker shades representing greater deviations.

To test this hypothesis further, we used our formal model to identify possible conditions that could interfere with maintenance of the prepattern proportions. When moving away from the region of boundary maintenance we previously identified in the expansion/division ratio space (Fig. 3C), the relative position of the boundary shifts from its initial condition because the two ratios are unbalanced. For instance, an increase in the proximal expansion rate is not compensated by a corresponding increase in the division rate of the distal region. Altering the growth ratio has a higher impact on pattern proportions than changing the division ratio; hence, the asymmetry when the ratios experience similar changes (in absolute values). To further explore configurations that could account for an upward shift of the final bullseye boundary in mature flowers, we singled out and analyzed the dynamics of two specific scenarios falling within the sub-region exhibiting a 0.2 increase in boundary position. In the first scenario, only the expansion ratio increases (case 3, Figs. 3C and 6A) while in the second scenario, both the expansion and division ratios are raised (case 4, Figs. 3C and 6A). Examining the impact of these scenarios on cell number and cell length ratios, we found that increasing the growth rate ratio alone shifts the boundary position and the length ratios upward (case 3, Figs. 3C and 6A), while the number of cell ratio remains similar to WT (compare case 1, Fig. 3D, with case 3, Fig. 6A). Increasing both the division rate and growth rate ratios (greater growth in the proximal region) shifts the boundary position and the number-of-cell ratio upward (case 4, Figs. 3C and 6A), while the length ratio remains similar to WT (compare case 1, Fig. 3D, with case 4, Fig. 6A).

To test whether petal morphogenesis in the *HtTCP4.1* OE line follows one of these two configurations, we extended our examination of cell behavior to the later phase of petal morphogenesis (S2L to S5)*.* Although the early pattern boundary in the *HtTCP4.1* OE transgenic line is established at one-third of the petal length (Fig. 5, C and D) as in WT, our analysis of subsequent developmental stages reveals that the relative position of the bullseye boundary rises to 0.5 at S2L before stabilizing around the 0.4 position at later stages (Fig. 6B). This corresponds to a 0.2 increase in boundary position, matching the one simulated in cases 3 and 4 (Fig. 3C). We found that petals of *HtTCP4.1* OE follow a similar morphogenetic process to WT: a phase of intense cell division followed by a period of cell expansion, with the distal cells exhibiting higher division rates during early development and proximal cells having higher effective growth rates (Fig. 6C). However, while the evolution of the cell length ratio (proximal/distal) is comparable to the one of WT *H. trionum*, the cell number ratio (proximal/distal) reaches a plateau at around 35% at S4, rather than 20% at S3 as observed in WT (Fig. 6D). While petals of both genotypes have a similar length, a detailed comparison reveals that both proximal and distal cells are overall longer in the *HtTCP4.1* OE line (Fig. 6E). In addition, there are more proximal cells but fewer distal cells (Fig. 6F) when *HtTCP4.1* is overexpressed, resulting in an overall lower cell number along the petal PD axis, accounting for the overall maintenance of petal length. These experimental conditions yield length ratios and number-of-cell ratios at maturity (S5) that do not match with the third scenario (case 3, Fig. 6, A and G), because this configuration shifts the boundary position but leads to a higher cell length ratio (Fig. 6C) and to a lower cell number ratio (Fig. 6D) than those experimentally recorded in the *HtTCP4.1* OE line. However, our experimental data align better with the outcomes theoretically predicted by the fourth scenario of our simulations (case 4, Fig. 6, A to G), with a log length ratio of 1.93, and a log cell number of 0.41 at maturity.

Together, our results suggest that the increased bullseye in *HtTCP4.1* OE follows the second theoretical mechanism outlined earlier, where the early boundary is specified one-third from the petal base, as in WT but with subsequent changes in growth (increase in cell division and expansion), shifting the boundary position upward and producing flowers with a larger bullseye.

### Bumblebees can discriminate targets solely based on bullseye size

**Fig. 7.**
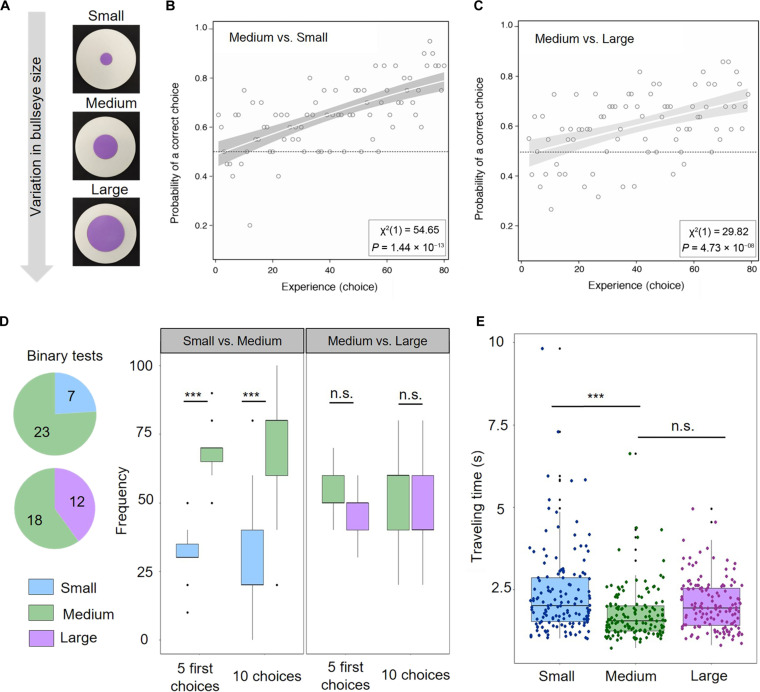
Bumblebee (*B. terrestris*) responses to varying bullseye sizes. (**A**) Epoxy discs featuring small (*H. richardsonii*–like), medium (*H. trionum* WT-like), and large (*HtTCP4.1* OE-like) bullseyes. Purple centers represent 4, 16, and 36% of the total area, respectively. (**B**) Learning curve of 20 individuals choosing between discs with small or medium bullseye sizes. Empty circles depict the mean proportion of bees choosing correctly for 80 successive choices. The white curve represents the fitted binomial logistic model, with gray shading indicating 95% confidence intervals on the fitted response. The χ^2^ statistic (the number in brackets indicates df) and *P* value for the likelihood ratio test (assessing whether foragers can learn) are provided. (**C**) Learning curve of 22 individuals choosing between discs with medium or large bullseye sizes. Similar annotations as in (B) are included. (**D**) Preference test experiments. See statistics in fig. S8. Binary test—number of naïve bumblebees choosing to first land on a disc with a small versus medium bullseye (top pie chart) or on a disc with a medium versus large bullseye. Bumblebees showed a statistically significant preference for the medium bullseye size compared to the small one. *n* = 30 bumblebees for binary tests. Ten choice tests: (left) when the first 5 choices or the first 10 choices were considered, bumblebees showed a statistically significant preference for the medium bullseye size compared to the small one; (right) when the first 5 choices or first 10 choices were considered, bumblebees showed no significant preference for the medium versus the large bullseye (one-sample *t* test). *n* = 15 bumblebees. (**E**) Distribution of individual travel time between discs for the three bullseyes size. *n* = 15 bumblebees for each bullseye size; each bumblebee flew 10 times between each disc type. Each dot corresponds to the flying time between two discs for each bee on each travel path.

Petal patterns are believed to enhance flower attractiveness and help visiting animals form a search image to identify targets effectively (*2*). To investigate whether pollinators can discriminate between Hibiscus bullseyes of different sizes and exhibit any innate preference for specific pattern proportions, we conducted experiments with naïve buff-tailed bumblebees (*B. terrestris*), known to pollinate Australian and New Zealand plants (*34*, *35*). We created 3D-printed discs that mimicked the color patterns of Hibiscus bullseyes. These discs were designed to match the dimensions of open flowers and differed only in the size of their pigmented area, replicating the bullseye proportions of *H. trionum* WT (medium bullseye), *HtTCP4.1* OE (large bullseye), or *H. richardsonii* (small bullseye) flowers with pigmented area accounting for 16, 36, and 4% of the disc surface, respectively (Fig. 7A). This allowed us to assess bumblebees’ response to variations in bullseye size when no other stimulus is present.

To familiarize bumblebees with our foraging setup, we initially trained them to feed on black artificial discs containing a 15% sucrose solution. We then conducted differential conditioning experiments to investigate their ability to distinguish between different bullseye sizes by assessing whether they could learn to associate specific proportions to the presence or absence of a reward. First, we randomly arranged five small bullseye discs (resembling *H. richardsonii*) and five medium-sized ones (resembling *H. trionum*) in the flight arena. We assessed the behavior of 20 individuals, with 10 offered a 20% sucrose solution (reward) on the medium-sized bullseye flower, and water (neutral) on the small-sized one, and the other 10 offered the opposite combination. Discs were refilled, and their positions randomized throughout the experiment. After 80 visits, bumblebees chose the rewarding discs (sucrose solution, correct choice) much more frequently than at the beginning of the experiment [learning curve (χ^2^ = 54.65, *P* < 0.001) (Fig. 7B)].

Initially, individuals visited the first 10 discs randomly [55% of correct choices, 95% confidence interval (47.4 to 63.6%)], but after 80 visits, the probability of making a correct choice (reward) significantly increased [82% of correct choices, 95% confidence interval (78.0 to 87.0%), *P* = 5.57 × 10^−12^]. This indicates that bumblebees can discriminate between small and medium bullseyes solely based on size. When examining individual behaviors, we noticed that 5 of 20 individuals already displayed a preference for the medium-sized bullseye (*H. trionum*–like) at the beginning of the experiment (70 to 90% correct choices during the first 10 visits), and the reward was consistently associated with medium bullseye in these cases (fig. S8A). Only two individuals showed no evidence of learning to distinguish between the two bullseyes (probability of correct choice during the last 10 visits = 0.6), and in both cases, the reward solutions were associated with the smaller, *H. richardsonii*–like pattern. This suggests that naïve bumblebees may have an innate preference for the WT *H. trionum* bullseye dimensions over the smaller, less conspicuous *H. richardsonii*–like pattern.

To determine whether bumblebees could differentiate between medium and large bullseyes, we conducted a similar experiment using 3D-printed discs featuring medium (*H. trionum*–like) and large (*HtTCP4.1* OE-like) pigmented bullseyes (Fig. 7A). Analyzing the collective behavior of all 22 individuals tested (Fig. 7C), our results indicate that bumblebees were capable of learning which type of bullseye was associated with a reward (χ^2^ = 29.82, *P* < 0.001). Bumblebees randomly chose which disc to visit during their first 10 visits [50.5% of correct choices, 95% confidence interval (44.4 to 56.5%)], but after 80 visits, individuals chose correctly (rewarding disc) almost three of four times [72.7% correct choices, 95% confidence interval (65.9 to 79.6%), *P* = 8.39 × 10^−7^]. However, learning to discriminate between medium and large bullseye sizes appeared to be more difficult for bumblebees than distinguishing between medium and small patterns. When analyzing individual behavior, half of the 22 individuals were able to associate bullseye size with the presence/absence of a reward (χ^2^ = 54.79, *P* < 0.001; fig. S8B), while the other half did not (χ^2^ = 1.74, *P* > 0.5; fig. S8C). Altogether, these results suggest that, on average, it may be more challenging for bumblebees to discern the size difference between a medium (WT *H. trionum* pattern) and a large bullseye (*HtTCP4.1* OE pattern) compared to the medium versus small bullseye combination. However, for those individuals that successfully differentiated medium patterns from large ones, their performances matched those of bumblebees asked to distinguish between medium and small bullseyes (compare Fig. 7B with fig. S8B).

### Bumblebees prefer the WT *H. trionum* bullseye proportion over its close relative *H. richardsonii*

Next, we used a binary choice experiment to test whether naïve bumblebees display an innate preference for specific bullseye proportions. Two equally rewarding discs (20% sucrose solution), one displaying a small bullseye and the other presenting a medium bullseye, were positioned equidistant from the hive entrance, and a single naïve forager was allowed to enter the arena, with its first choice recorded. Of 30 bumblebees, 23 chose to land first on the medium-sized bullseye disc (Fig. 7D and fig. S8D). To evaluate whether this preference persisted during a foraging bout, we randomly placed five artificial discs of both bullseye sizes across the arena, all containing the rewarding solution. We recorded the first 10 choices made by 15 naïve bumblebees, refilling and changing flower position as the individuals foraged. Whether considering the first 5 or first 10 choices, bumblebees consistently preferred the medium-sized bullseye (WT *H. trionum*) over the smaller one (*H. richardsonii*). Specifically, the *H. trionum*–like discs were chosen 7 of 10 times (Fig. 7D and fig. S8D). We repeated this experiment using medium (WT *H. trionum*) and large (*HtTCP4.1* OE) bullseyes. In this case, regardless of whether we considered the binary choice, first 5 choices, or all 10 choices, we could not detect any statistically significant preference for either of the two bullseye sizes (Fig. 7D and fig. S8D).

### Enlarged bullseye size enhances flower detection

To investigate the possible impact of bullseye size on flower detectability, we recorded the time individuals took to move from one target to the next (foraging speed) for each of the three types of pattern dimensions. We found that bumblebees flew significantly faster (*P* = 0.0023) between artificial flowers displaying a medium-sized bullseye (WT *H. trionum*) compared to those foraging on discs with a smaller pattern (*H. richardsonii*) (Fig. 7E). However, no significant difference in mean travel time was observed when comparing large bullseyes (*HtTCP4.1* OE) to medium-sized ones (*P* = 0.23), consistent with our previous findings, indicating that bumblebees may find distinguishing between those two bullseye sizes challenging. Overall, our data indicate that foragers can discern between targets solely based on bullseye size differences and use pattern dimensions as a reliable cue to identify rewarding flowers. Our findings also demonstrate that bullseye size directly impacts flower detectability and that buff-tailed bumblebees exhibit a strong innate preference for *H. trionum* bullseye over the smaller pattern of its close relative, *H. richardsonii*.

## DISCUSSION

Our analysis of developing *H. trionum* petal primordia revealed that cell behavior across the adaxial epidermis is prepatterned, characterized by regional differences in cell expansion and division along the base-to-tip axis (PD axis) long before a visible bullseye emerges. Following an initial stage of uniform behavior (S0a), the distal petal mainly grows through cell division, while the size of the proximal region increases predominantly through cell expansion. From stage 0b onward, the largest cells are concentrated in a region invariably positioned one-third from the petal base. These cells could represent the first cells to initiate differentiation across the adaxial petal epidermis, becoming anisotropic by elongating preferentially along the PD axis. This landmark also corresponds in later stages to the transition point between pigmented and nonpigmented cells that characterize the final bullseye boundary. Hence, the prepatterning phase may already specify bullseye boundary cells early on, influencing pattern proportions. However, further investigations are needed to determine whether the largest cells emerging during the prepatterning phase are indeed the first to differentiate and act as progenitors for the bullseye boundary cells. Regardless of whether the early pattern boundary yields the final bullseye boundary, our results show that the partitioning of the adaxial epidermis into subdomains during early petal development influence the emergence of distinct cell behaviors in neighboring regions. An early pattern boundary specified closer to the petal base is associated with the production of a smaller bullseye in *H. richardsonii*. The pigmented area in *H. richardsonii* is even smaller than the proximal domain. This indicates that although the shift in early boundary specification we uncovered along the petal PD axis is sufficient to produce the smaller structural bullseye (i.e., flat tabular striated cells cover a smaller portion of the total petal area), additional changes affecting gene(s) controlling pigment production, acting downstream of those controlling the prepatterning process, must also have occurred along the lineage leading to *H. richardsonii* during evolution.

While our study focuses on *H. trionum* and its closest relative, prepatterning the petal epidermis along the PD axis likely represents a general mechanism shared by a multitude of species. For instance, a Turing-like process was recently proposed to produce the spotted patterns on the ventral petals of *Mimulus lewisii* and *Mimulus guttatus*. The suggested mechanism relies on a tug-of-war between two TFs from the MYB family: NEGAN (NECTAR GUIDE ANTHOCYANIN), an activator of anthocyanin pigment production (*17*), and RTO (RED TONGUE), a repressor of NEGAN (*12*). This model is particularly elegant as it adheres to the principle of self-organization (*36*) and does not require the existence of an early pattern. However, in natural variants or knockout lines where RTO activity is absent, the spots are replaced by uniform pigmentation. This pigmentation does not extend to the entire petal epidermis but remains confined to the proximal part of the petal, resembling a red tongue. This suggests that, in addition to the spotted phenotype generated by a Turing-like system, the petal epidermis is also compartmentalized into distinct domains along the PD axis. The absence of RTO activity removes pigment production repression within one of those domains (the proximal region) and renders the existence of petal compartments apparent. To further explore this hypothesis, it would be valuable to investigate whether the identity of the future yellow distal and red proximal regions of the Mimulus petal are also specified during a prepatterning phase similar to what we uncovered in Hibiscus.

Prepatterning may represent an ancestral process plants used to specify cell fate along the different axes of their lateral organs long before flower originated. A prepattern mechanism has been proposed to play a role in establishing abaxial-adaxial polarity in leaves, with spatial information provided by the activities of REVOLUTA, AS2, and KANADI1 across the shoot apical meristem, positioning the adaxial auxin response (*37*, *38*). More relevant to our investigation, a proximodistal gradient of gene expression has recently been suggested to establish a prepatterned transcriptomic boundary in maize leaf primordia (*39*). This gradient exists within morphologically indistinct structures, i.e., before the morphological changes that define the blade and sheath domains, suggesting that a gene expression prepattern sets a developmental boundary along the proximodistal axis of young maize leaves. From an evolutionary viewpoint, petals can be viewed as modified leaves; thus, it will be important to identify the molecular players orchestrating petal patterning and test whether those differ from the agents responsible for leaf patterning.

While the mature bullseye at S5 exhibits clear bilateral symmetry, the early development of the petal epidermis surprisingly reveals a right-left asymmetry. Initially, larger cells first emerge near the attachment point to the floral structure and the ovary base on one side of the petal. Notably, the thickness of the petal base is uneven, and the early pattern consistently initiates from the thicker side that likely has a stronger connection to the rest of the floral structure. This suggests that an upstream positional signal, produced externally, could be responsible for prepatterning the petal epidermis, with the cells closest to the source of this signal being the first to modulate their behavior. Not only does the early pattern arise from one side, but the emergence of pigmentation is also asymmetric. Coloration initially appears as two dots on either side of the petal attachment point at S1 (Fig. 1B), yet the pigmented mark associated with the thicker side of the petal base always forms earlier and often appears larger. This reinforces the idea that prepatterning of early cell behavior and the development of bullseye features are closely intertwined.

Flower patterns come in diverse types (stripes, spots, bullseye, etc.) but also exhibit variations in dimensions. Here, we explored the mechanisms contributing to the variation in bullseye proportions. We conducted a comparative analysis in flowers with different bullseye sizes: a transgenic line overexpressing *HtTCP4.1*, resulting in a larger bullseye compared to WT *H. trionum*, and a close relative of *H. trionum*, *H. richardsonii*, which exhibits a significantly smaller bullseye. In *H. richardsonii*, the peak of larger cells first becomes apparent at S0c, positioned around 0.15 to 0.2 from the petal base (in contrast to 0.3 for *H. trionum*). This indicates a downward shift of the prepattern boundary along the petal PD axis. From S2 onward, the peak of larger cells remains closer to the base (around the 0.1 to 0.2 position), aligning with the position of the boundary typical of the smaller bullseye of *H. richardsonii*. These findings support the idea that (i) the early pattern could determine final bullseye dimensions, and (ii) early pattern proportions can be maintained while the petal grows to S5, allowing the bullseye to scale up along with flower size. In contrast, early boundary emergence from S0a to S1 in the *HtTCP4.1* OE line is similar to WT, implying that the significant increase in bullseye dimensions observed in mature flowers is not due to a shift in early pattern boundary positioning. Instead, both computational simulations and experimental observations support the idea that this larger bullseye is due to later changes in growth, acting as pattern modifiers. At S2, cellular behavior diverges from WT, with a higher proportion of large cells spreading around the 0.2 to 0.5 position. Unlike WT petals where cell division and DNA replication are mainly restricted to the distal region, cell proliferation events occur across the entire S0a petal adaxial epidermis when *HtTCP4.1* is overexpressed. This suggests that the larger bullseye observed in the *HtTCP4.1* OE line results from an excess of cell proliferation at the petal base early in development (S0a), positioning more cells to acquire the fate of the proximal domain (i.e., change in initial conditions). Proximal cells, programmed to grow more than the distal ones during the differentiation process, lead to larger cells at the petal base, ultimately resulting in a larger bullseye. Together, these results illustrate how local variations of growth and cell proliferation on either side of the early pattern boundary can act as a robust mechanism for modulating pattern proportions and thereby regulate the dimensions of the final bullseye. Thus, partitioning the petal epidermis into subdomains not only plays a role in controlling cell fate specification spatially but also constitutes an effective system for autonomously regulating growth in two neighboring domains of the epidermal tissue, where DNA replication, cell division, and expansion are independently controlled.

Hormonal cross-talk, especially the balance between auxin and cytokinin, is central to the patterning of Arabidopsis roots, ovary, and grass leaves (*40*–*42*). Considering the well-known roles of both hormones in regulating cell proliferation and expansion, it is likely that plant hormones also contribute to the control of bullseye dimensions. For instance, TCP4 in Arabidopsis has recently been shown to promote auxin synthesis during development via transcriptional activation of *YUCCA5* expression (*43*). *HtTCP4.1* is preferentially expressed in the distal portion of the *H. trionum* petal, where most cell division events occur, and ectopic overexpression of *HtTCP4.1* is sufficient to induce excessive cell proliferation in the proximal region at S0a. These observations suggest that HtTCP4.1 participates in setting the bullseye dimension by promoting cell proliferation in the distal domain and that manipulating its spatiotemporal expression constitutes a means to modify bullseye proportions using a pattern modifier process. Whether HtTCP4.1 activity in Hibiscus relies on local activation of auxin production will need to be tested in further studies.

We found that bumblebees can effectively distinguish between medium and small bullseyes mimicking those of *H. trionum* and *H. richardsonii*, respectively, based on size differences only. Even without a strong incentive [without using quinine hemisulfate salt solution as punishment (*21*), but rather a neutral solution of water against a rewarding sugar solution], bumblebees successfully discriminated between the two bullseye sizes, indicating that they can easily detect the difference. Preference and binary choice tests further revealed that buff-tailed bumblebees have an innate preference for *H. trionum*–like bullseye size over the smaller pattern of its relative *H. richardsonii*. Foraging tests also showed that bumblebees could detect targets faster when a medium rather than a small bullseye was present on the discs. Further investigations are required to determine whether this preference holds in a more realistic context, when additional elements like UV or scent might compensate for the reduction in pigmentation, potentially affecting overall attractiveness. However, our results indicate that flowers with reduced bullseyes could be discriminated against when growing alongside flowers with larger patterns. Notably, *H. richardsonii* is classified as a “vulnerable” species in Australia and as “threatened/nationally critical” in New Zealand (*24*), with populations declining in their natural habitat. While the exact causes remain uncertain and are likely to be multiple, the reduction in bullseye size may contribute to a decline in pollinator attraction. Our behavioral experiments have focused on buff-tailed bumblebees, and the response of other foraging insects could differ. One intriguing hypothesis is that a change in bullseye dimensions could mediate a change in pollinator type. Whether a change in pattern proportions can lead to reproductive isolation and promote speciation are open questions that will certainly necessitate field investigations.

**Fig. 8.**
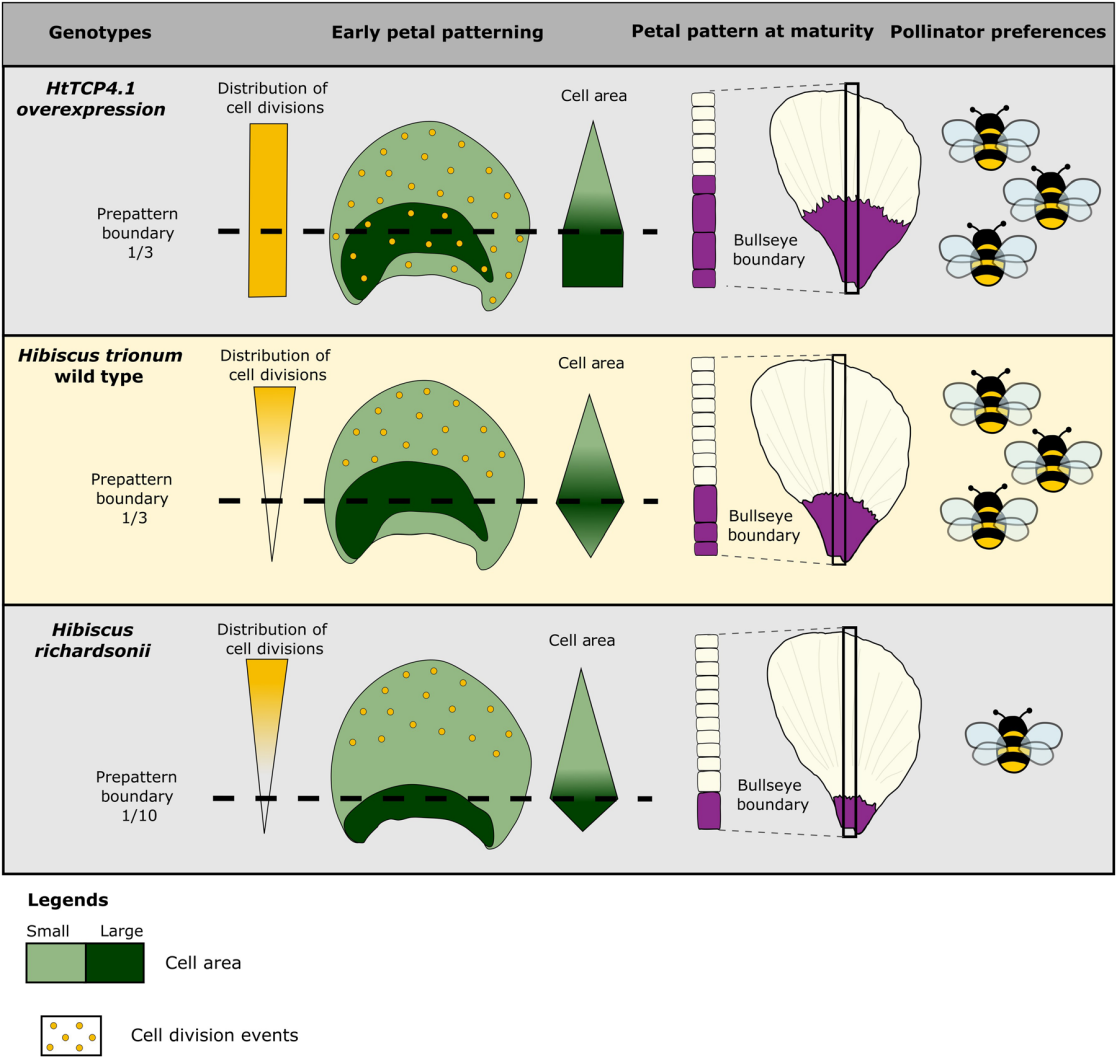
Summary of processes involved in setting up bullseye pattern proportions and its impact on bumblebee behavior.

To conclude, our study highlighted that the establishment of a prepattern is a key feature of Hibiscus petal development. Events affecting the patterning process itself early in development (modification of the early pattern boundary position) or processes acting as pattern modifiers at later stages (local change in growth/cell division either side of the boundary) represent two distinct mechanisms equally able to produce variations in bullseye proportions (Fig. 8). Such modifications in pattern dimensions hold crucial biological importance, as buff-tailed bumblebees can distinguish flowers based on bullseye size only and exhibit an innate preference for medium-sized patterns over smaller ones. What genetic bases account for differences in bullseye size between the two sister species and whether such a change in pattern dimension contributed to reproductive isolation and speciation represent interesting venues for future research.

## MATERIALS AND METHODS

### Plant material

*H. trionum* L. seeds were originally obtained from Cambridge University Botanic Garden; *H. richardsonii* seeds [Mayor Island (Tuhua), New Zealand—Voucher AK251841] were supplied by B. G. Murray (*44*). WT *H. trionum* and *H. richardsonii* plants and *35S::HtTCP4.1* or *35S::HtTCP4.2* transgenic *H. trionum* lines were grown under glasshouse conditions on a 16-hour:8-hour, light: dark photoperiod at 23°C in Levington’s M3 (UK) compost.

### Production of the *HtTCP4.1* and* HtTCP4.2* OE lines

Gibson assembly (*45*) and primers oEM252-F (AGGATCCACTAGTCAAGTTTGAATTCATGGGAGATAGCAATCACC), oEM253-R (GGTGATTGCTATCTCCCATGAATTCAAACTTGACTAGTGGATCCT), oEM254-F (CTCTGACTCTCACCATTGAGAATTCATGCTAGAGTCCGCAAAAAT), and oEM255-R (ATTTTTGCGGACTCTAGCATGAATTCTCAATGGTGAGAGTCAGAG) were used to insert the full-length coding sequence of *HtTCP4.1* into a modified pGREEN II vector backbone containing a double 35S promoter (pEM110), yielding the plant expression vector pEM105. The coding sequence of *HtTCP4.2* was introduced into the same pEM110 backbone using the Bam HI and Hind III entry sites and primers oMY049-F (ATAAGCTTTAATGGGGGACAGCCAC) and oMY047-R (AGGGATCCCTTCAATGGTGAGAATCGGACGA), yielding the plant expression vector pMY40. The coding sequences of *HtTCP4.1* and *HtTCP4.2* have been deposited in GenBank under accession numbers OR908924 and OR985928, respectively. Transgenic *H. trionum* lines constitutively overexpressing *HtTCP4.1* or *HtTCP4.2* were obtained using pEM105 and pMY40, respectively, and Agrobacterium-mediated transgene delivery was followed by tissue culture to induce callus production and plant regeneration, following the protocol of Moyroud *et al.* (*25*).

### Confocal imaging and image analysis

#### Distribution of the cell features across the petal from S0a to S2E using confocal microscopy

*H. trionum*, *HtTCP4.1* OE, and *H. richardsonii* petals were dissected from S0a to S2E and mounted on a petri dish with double-sided tape. 3D depth-composition images of each petal were acquired using a Keyence VHX-7000 digital microscope at 100× to 300× magnification. Petals were stained with FM1-43 (0.01 μg μl^−1^; Thermo Fisher Scientific) for 15 min to 5 hours depending on their stages (respectively S0a and S2E) and incubated in the dark. Petals were washed twice with water before imaging using a 20× water-dipping objective on an LSM880 confocal microscope (Zeiss, Germany), excitation at 514 nm and emission filters set to 537 to 622 nm. For S0a to S1 primordia, the whole petal was imaged. Only the central stripe of the primordium was imaged in S2E petal. For each, multiple Z-stacks (2.5 μm spacing) were acquired to cover the whole surface. For each petal, image stacks were stitched using ImageJ (Version 1.53q). Images were then analyzed using MorphographX (*26*). The petal surface was extracted, and cell segmentation was performed, using the auto-segmentation function. Segmentation errors were corrected manually, and final meshes were converted into 2D meshes. The final template was processed with R and Matlab (Version 2020a) to extract the cell geometry information. To analyze the cell geometry distribution along the PD axis of the petal, a stripe of cells (20% of the width of the petal at its widest point) in the central region of the petal was analyzed.

#### Distribution of the cell features across the petal at S5

Images were acquired using a Keyence VHX-7000 digital microscope at 300× magnification. Cell features were manually measured along a line of cells, across the PD axis of the petal, using ImageJ (Version 1.53q) and processed using Matlab (Version 2020a).

#### Distribution of the cell division events across petals using EdU staining

For combined 5-ethynyl-2-deoxyuridine (Invitrogen A10044, Thermo Fisher Scientific) and modified pseudo-Schiff–propidium iodide (PI; Sigma-Aldrich) staining, the published method (*27*) has been modified. Buds from stage 0a to stage 1 were harvested and their sepals were removed before being embedded in the EdU staining medium [0.22% (w/v) Murashige and Skoog basal salts mixture, 3.5% (w/v) sucrose, 0.004% (w/v) l-cysteine, 0.0015% (w/v) ascorbic acid +0.01% myo-inositol (w/v) + 0.0001% nicotinic acid (w/v) + 0.0001% (w/v) pyridoxine hydrochloride +0.01% (w/v) thiamine hydrochloride +0.0002% (w/v) glycine +175 nm N6-benzyladenine +10 μM EdU (Invitrogen A10044, Thermo Fisher Scientific), pH 5.7] containing 0.8% (w/v) agarose. Liquid EdU staining medium was then added to immerse the bud. The samples were cultured for 13 hours in a growth chamber (16-hour light:8-hour dark photoperiod, average light intensity of 85 μM and average temperature of 24°C). Petals were the dissected from the buds and all petals were dehydrated by successive 15-min treatments in an ethanol dilution series (15, 30, 50, 70, 85, 95, and 100% EtOH) and stored in 100% EtOH overnight. Samples were rehydrated through the same EtOH series and incubated at 37°C overnight in alpha-amylase (Sigma-Aldrich A4551), phosphate buffer (0.3 mg ml^−1^; 20 mM), pH 7.0, 2 mM NaCl, and 0.25 mM CaCl_2_. Petals were washed twice in water and once with tris-buffered saline, pH 7.4, before being incubated for 1 hour in solution containing 10 μM Alexa 488-azide (Invitrogen A10266, Thermo Fisher Scientific) and 100 mM tris, pH 8.5; this was followed by 30 min in solution containing 10 μM Alexa 488-azide, 100 mM tris, 1 mM CuSO_4_, and 100 mM ascorbic acid, pH 8.5. Incubations were carried out at room temperature with gentle shaking and covered from the light. The samples were washed three times with water, treated in 1% periodic acid for 30 min, and washed twice with water, before being incubated in PI (0.01 μg μl^−1^) for 3 hours, covered with gentle shaking. The petals were cleared with a chloral hydrate solution for 2 hours and mounted in Hoyer’s solution (30 g of gum arabic, 200 g of chloral hydrate, 20 g of glycerol, and 50 ml of water). Samples were imaged with a Zeiss LSM880 imaging system with 20× objective lens. Excitation was at 488 and 561 nm; emission filters were set to 499 to 526 nm for EdU and 603 to 629 nm for PI. For each sample from stage 0a to stage 1, multiple 3D Z-stacks were acquired to cover the whole petal. For stage 2, only a central stripe was imaged. Images were then processed using Imaris 9.2.1 (Bitplane). A stripe of 100 μm was selected in the center of the petal and EdU-labeled nuclei were identified using the spot detection function (spot diameter: 4.16 to 5 μm). The data were then exported and analyzed using Matlab (Version 2020a).

### Statistical analysis

Graph and statistics were obtained with R, Matlab, or Excel software. Statistical tests performed are specified in figure legends. For mean area differences between regions of the PD axis in *H. trionum*, statistical differences have been calculated using the mean area of cells belonging to the [0.05;0.15[, [0.025;0.35[, and [0.8;0.9[ PD regions (see statistical results, Table 1). The homogeneity of cell area distribution at stage 0a between *H. trionum*, *HtTCP4.1* OE line, and *H. richardsonii* was analyzed using a two-sample Kolmogorov-Smirnov test, after verifying the normality of the data.

### Computational modeling

#### Data analysis

Boundary calculation: For the automatic calculation of the boundary between the two regions (used for the early pattern stages before the appearance of the pigmentation) from the data coming from MorphoGraphX in Fig. 5, we used the area as that is one of the defining characteristics for each cell type. For each sample, we first binned the cells in 5% windows based on their normalized position along the PD axis, *y*/*L*, where *L* is the length of the tissue, to calculate an averaged cell area along the PD axis. This window-averaged cell area is then further passed through a Savitzky-Golay filter to smooth the signal. Boundary position is then defined as the position of the average of all the windows within 2.5% of the window with the highest cell area.

Genotype comparison: To get the distribution of the ratios of the areas, number of cells, and boundaries, we use the following definitions of the mean and variance of the ratio of two random variables, *X* and *Y*

E(X/Y)=E(X) E(1/Y)

$$Var\left(X/Y\right)=E\left(X^{2}\right)E\left(1/Y^{2}\right)-E^{2}\left(X\right)\;E^{2}\left(1/Y\right)$$

The error bars on the plots are the sample standard deviations, $\sqrt{\text{Var}\left(X/Y\right)}.$

#### Model description

Because the main question in this part of the work is the positioning of the boundary between cell types along the PD axis and our data are also mainly given in terms of their positioning along this axis, we represented the petal as a 1D array of cells. This is a simplification, and it has been done for other tissues where one dimension of the tissue dominates, for example, when considering hormone distributions in the root (*28*). The cells then grow and divide in a way that depends on their type and current size.

We used the modeling language Chromar, which has been used before in cell-based simulations (*46*). Chromar uses discrete objects that carry attributes to represent entities in the model and rules on these objects to describe the dynamics. Rules are stochastic and the simulation is done with a version of the stochastic simulation algorithm (Gillespie—we refer the reader to the paper describing the language for details). In this case, we used an implementation of this framework in Python (code deposited on Dryad DOI: 10.5061/dryad.f4qrfj745). Rules can also use aggregates over the state of the system called observables. Observables are made from two parts, a “selection” part where one specifies which objects to choose from the state (e.g., all cells that have fate 0), and an aggregation part that defines how to reduce these cells into a single value (e.g., sum the length of the chosen cells).

In this case, our objects are cells that have the following type

$$\text{Cell}\left(\text{cid}:\text{int},\text{neigh}:\text{int},\text{type}:\left\{0,1\right\},\text{len}:\text{float}\right)$$

where cid is an integer cell identifier, neigh is an integer cell identifier representing the identity of their right neighbor, type is identifier for their type (fate), which, in this case, we choose to be either proximal or distal, and, lastly, len is a float number representing their length. For notational convenience, we use 0 for the type “distal” and 1 for type “proximal.”

For growth, we have the following rule

$$\begin{array}{c} \text{Cell}\left(\text{len}=l,\text{type}=f\right)\\ \to \text{Cell}\left[\text{len}=r+r_{g}\left(f\right)\;l\left(1-\frac{l}{l_{max}}\right),\text{type}=f\right] \end{array}$$

thus, any cell in the state can grow its length by some amount that depends on its current length, its type [*r_g_*(*f*)], and how far away this length is from a maximal length that the cell can take (*l*_max_). The last two are functions of its type (*47*).

For division, we have the following rule

$$\text{Cell}\left(\text{cid}=i,\text{neigh}=n,\text{type}=f,\text{len}=r\right)\;\to_{d(f,L)}$$

$$\begin{array}{c} \text{Cell}\left(\text{cid}=i,\text{neigh}=\mathrm{nc}+1,\text{type}=f,\text{len}=r/2\right),\\ \text{Cell}\left(\text{cid}=\mathrm{nc}+1,\text{neigh}=n,\text{type}=f,\text{len}=r/2\right) \end{array}$$

where

$$d\left(f,L\right)=r_{d}\left(f\right)\;e^{-L/L_{0}\left(f\right)}$$ (1)

thus, any cell can divide at a rate *d*(*f*, *L*) that depends on its fate and the current length of the whole petal. This rate depends on a basal division rate that depends on the fate of the cells, *r_d_*(*f*), and a factor *e*^−*L*/*L_o_*(*f*)^ that depends on the current length of the entire petal and a function of its type, *L_o_*(*f*), that controls the timing of a slow-down in the division rates that is fate dependent. This slow-down of divisions over time is used to create the expansion phase of the development of the petal as observed in the data. Because the length of the petal is not in the attributes of the cell on the rule’s left-hand side, it needs to be computed with an observable

$$\begin{array}{c} L=\text{select}\;\left[\text{Cell}\left(\text{cid}=i,\text{neigh}=n,\text{type}=t,\text{length}=l\right)\right];\\ \text{aggregate}\;\left(Lacc:\text{float}.\text{Lacc}+l\right)\;0.0 \end{array}$$

A division gives rise to a new cell to the right of the newly divided cell. To be able to give fresh ids to the new cells, we need to know how many cells there are in total. Thus, we lastly have the following observable to keep track of the number of cells in the tissue so we can give fresh ids to newly created cells

$$\begin{array}{c} nc=\text{select}\;\left[\text{Cell}\left(\text{cid}=i,\text{neigh}=n,\text{type}=t,\text{length}=r\right)\right];\\ \text{aggregate}\;\left(count:int.count+1\right)\;0 \end{array}$$

#### Model analysis

Initial and final state: For convenience for the initial state, we started with 21 cells of length 0.1 μm, giving an initial length of around 2 μm. From these cells, the first seven have the proximal fate and the rest have the distal fate. Thus, this assumes that although there is no visible difference in the cell morphology, the cells have already assumed a fate in the beginning of the simulation and that the position of the fates along the PD axis is at the one-third position.

In the data, we observe an increase in total length from 200 μm in stage S0a to about 30,000 μm in S5. For the model then, we use a similar increase of ~×150 for a target total length of the petal of 300 μm (Parameters, Table 2). The scaling down of the petal in terms of the number of cells in the simulation is done for computational efficiency reasons while not affecting the conclusions of the model.

Parameters: Plotting the average growth dynamics of cell numbers and sizes (Fig. 5) allowed us to get a quantitative appreciation of the dynamics. To analyze the effect of model parameter values, we first performed a more careful estimation of the growth parameters to fit with the data. Parameters used for the model, unless otherwise stated, are summarized in Table 2.

**Table 2.** Main parameter values for the simulation in Fig. 6.

Parameter	Values	Description
*r_g_*(0)	0.91 (μm/day)	Base growth rate of distal cells; the base growth rate of proximal cells is given in reference to this
*r_d_*(0)	1.1 (1/day)	Base division rate of distal cells; the base division rate of proximal cells is given in reference to this
*l*_*max*_	20	Maximum length of cells
*L*_0_(0)	0.1*L_max_	Half-point for the decline in division rates as a function of the “target” length of the petal. For the proximal fate, this is given in terms of the distal *L*_0_.
*L*_*max*_	300	Threshold total length of petal
*L*_0_(0)/*L*_0_(1)	0.6	Difference in timing of exit from division-heavy phase for the two fates

For the growth and division parameters, (*r*_*g*_,*r*_*d*_), we estimate the rate for the distal cells from the data and then define the rate for the proximal cells relative to this value. The number of cells can be approximated to increase exponentially between states (Fig. 3F), giving an equation for the number of distal cells *dN*_0_/*dt* = *kN*_0_, with solution *N*_0_(*t*) = *N*_0_(*t*_0_)*e^kgt^*, where *N_o_*(*t*_0_) is the initial number of distal cells and *N*_0_(*t*) is the number of distal cells at time *t*. The rate at time *t_i_* (a specific stage) is calculated as the rate of growth from the previous time *t*_*i*−1_ (the previous stage) as *k*_*t*_*i*−1_→*t_i_*_ = ln [*N*_0_(*t_i_*)] − ln [*N*_0_(*t*_*i*−1_)]/(*t_i_* − *t*_*i*−1_). The timings of stages used in this calculation are given in the “Estimation of the stage durations” paragraph below and the number of cells for each stage is the median of the cell numbers for that stage across the samples. No rate calculation was made for the first stage, S0a. Plotting the estimated rates against the length of the petal gives us an estimation of the parameters in *d*(0, *L*) (Eq. 1 and fig. S3A), which are approximated using an exponential function.

Cell growth rates are harder to estimate because they are a combination of both an inherent “real” growth rate of the cells and the rate of division. We can, nevertheless, assuming an exponential growth as above (fig. S3B), estimate the effective growth rate of cells as *k*_*t_i_*→*t*_*i*+1__ = ln [*l*(*t_i_*)] − ln [*l*(*t*_*i*−1_)]/(*t_i_* − *t*_*i*−1_), where *l*(*t_i_*) is the median cell area for all the cells for that stage across samples. The timings used for the stages are given in the “Estimation of the stage durations” paragraph below, and as before, there is no calculation for the first stage, S0a. The effective growth rate should be at its closest to the real growth rate toward the end of the process when the effect of divisions is at its lowest; we therefore used the calculated final effective growth rate as the true basal growth rate of the distal cells, *r_g_*(0). The parameters were also adjusted to daily instead of hourly rates to decrease the number of events in the simulations. Despite the uncertainty over the exact value of the true growth rates of cells in the regions, conclusions from the model are about ratios of parameter values and still hold even when the absolute values are changed by up to 20% (fig. S3C).

Estimation of the stage durations (approximation for stages 0a-0b-0c): The timing for each stage is the following: S0a, 1 hour; S0b, 31 hours; S0c, 62 hours; S1, 93 hours; S2E, 152 hours; S2L, 211 hours; S3, 309 hours; S4, 404 hours; S5, 416 hours.

#### Parameter analysis

We have quantified the effect of the varying the ratios of the growth characteristics of the two domains in Fig. 3. To quantify the extent of the effect of the other parameters of the model (Table 2), we used the ratio of the relative percentage change of the observable to the relative percentage change of the parameter

$$\frac{\left[obs\left(\theta \prime \right)-obs\left(\theta \right)\right]/obs\left(\theta \right)}{\left|\theta \prime -\theta \right|/\theta }$$

Thus, a value of this sensitivity below 1 means that the observable changed less in percentage than the parameter, and a value of more than 1 means there was more percentage change in the observable relative to the parameter. We focused on the effect of small parameter changes (+10%, −10%) from the parameters in case 1 on the three main observables (fig. S4A). Because these parameters appear in the growth terms of the rules, the biggest changes are in the length ratio and number of cell ratio observables. The boundary position is less affected with changes in length ratio balanced by number of cell changes to keep the relative areas of the two domains unaffected.

Last, by changing the initial conditions so that the boundary between the two domains is at different positions along the PD axis, we showed that our conclusions about the conditions leading to boundary position maintenance (case 1, Fig. 3D) are not specific to the one-third position chosen in the main results presented (fig. S4B).

#### Simulations

For each parameter choice referred to in Figs. 3 and 6 and figs. S3 and S4, what changes is the ratio of the specific values of the basal growth rates per cell fate *r_g_*(0), *r_g_*(1) and *r_d_*(0), *r_d_*(1). All the simulations are run until the tissue reaches a certain threshold length. The total length of the tissue is the sum of the lengths of all the cells

$$\text{tl}=\sum_{i}l_{i}$$

Because each simulation is stochastic for every parameter choice, the model was run 50 times, then each of the interesting observables (number of cells in each region, growth rates in each region, etc.) was binned into 1% of simulation-time windows to get 100 numbers per observable. These were then averaged across the 50 simulations.

#### Objective functions

To probe the behavior of the model, we used the dynamics of the position of the boundary, the area ratio—the ratio of average cell length in the two fates—and the number of cell ratio—the ratio of the number of cells in each fate as outputs. To calculate these from the state of the model, we define the following observables for the number of cells in each fate, nc0 and nc1, and the total length of each region, tl0, for the total length of the distal region and tl1 for the total length of the proximal region

$$\text{nc}0=\left|\left\{i|f_{i}=0,1\leq i\leq n\right\}\right|$$

$$\text{nc}1=\left|\left\{i|f_{i}=1,1\leq i\leq n\right\}\right|$$

$$\text{tl}0=\sum_{\left\{\left.i\right|f_{i}=0,1\leq i\leq n\right\}}l_{i}$$

$$\text{tl}1=\sum_{\left\{\left.i\right|f_{i}=1,1\leq i\leq n\right\}}l_{i}$$

where *f_i_* is the fate of cell *i*, *l_i_* is the length of cell, and *n* is the total number of cells. The area ratio then is $\frac{tl1/nc1}{tl0/nc0}$ , the number of cell ratio is $\frac{nc1}{nc0}$ , and the boundary position is $\frac{tl1}{tl1+tl0}$ . These observables are calculated per step in the model output, binned into 1% of simulation-time windows and averaged to get 100 numbers. These were then averaged across 100 simulations. Because the length of the petal is a more accurate description of development and to ease the comparison to the experimental data, these were then resampled to every 10% of final length of the simulated petal to the nearest time percentage. Thus, for example, the boundary position at 10% petal length (30, because the target length is 300) would be the value of the boundary position observable (calculated as above) at the time window where the length of the petal was nearest to that 10% of the total petal length.

To compare the model observables, the same observables had to be calculated for the experimental data. The calculated observables (Fig. 5) were interpolated and sampled to every 10% of final petal length as the model observables. Last, to compare a simulation to a data observable, we used the mean percentage error. Thus, if the value of the model observable at 10% petal length is *obs*_*sim*_ and the value of the data observable at 10% petal length is *obs*_*data*_, the percentage error is (*obs*_*sim*_ − *obs*_*data*_)/*obs*_*data*_. These are then averaged across all samples. The total error for all three observables is the average of the three. This is what is plotted in Fig. 6C in the main text.

### Behavioral experiments

Flower-naïve bumblebees (*B. terrestris* var. audax) (Research Hive, UK) experiments were conducted using the same arena design as previously published (*21*). In total, in this study, foragers from three colonies were used. Each colony was fed daily with fresh 15% sucrose solution and twice weekly with pollen (The Happy Health Company or Biobest). To distinguish individuals during experiments, foragers were hand-marked with water-based Thorne queen marking paint of various color associations.

#### Artificial flower design

All artificial disks were 6 cm in diameter, made with a 3D printer, using polylactic acid filament (1.75 mm) and fixed, using Velcro dots to a 3-cm cylinder of green FIMO polyester clay. For the training phase, uniform black disks were used. For all differential conditioning, preference tests, and foraging speed experiments, disks were bi-colored with an outer white ring surrounding an inner purple circle of variable sizes depending on the desired bullseye dimensions: 1.2 cm (4%) in diameter for the small bullseye (*H. richardsonii*–like), 2.4 cm (16%) in diameter for the reference bullseye (medium size, *H. trionum*–like), and 3.6 cm (36%) in diameter for the larger bullseye (*35S::HtTCP4.1*-like).

#### Training phase

To familiarize themselves with the foraging setup, bumblebees foraged freely from seven black artificial flowers (training discs), randomly positioned in the arena, each of them containing 45 μl of 15% w/w sucrose solution. After feeding, discs were removed, cleaned with 70% ethanol, and replaced in a new position. A bee was considered trained after it has made several return trips from the arena to the hive.

#### Differential conditioning experiments

During the test phase, two types of discs were compared at a time. Five artificial flower disks of one type (either small or large) plus five of the medium bullseye sizes were randomly positioned in the arena. Only one type of disc contained 45 μl of 20% sucrose solution (reward), the other type displayed 45 μl of ddH_2_O water (neutral reward). An individual trained bee was released into the arena, and the discs that it visited were recorded. A disc was considered visited whenever a bee landed on it. After each visit, the disc was refilled with sucrose or water solution, and its position changed. Discs with which the bee came into contact were cleaned with 70% ethanol between each foraging bout and individual bees. For each bullseye size combination, experiments were performed with 20 bumblebees in total: e.g., for the comparison small versus medium size, reward was presented on 10 bumblebees on the small disc and reward was presented on the medium for 10 other ones. Each bee was tested up to a minimum of 80 choices. Statistical analyses were performed using RStudio (Version 1.1.1717). Learning curves associated with each pairwise comparison were obtained by pooling data from individual bees, as described in (*21*).

#### Preference tests

Two types of preference tests were performed. The first one was a binary choice experiment, where a naïve bumblebee was presented with two discs at equidistant entrance of the hive and equally rewarding (45 μl of 20% sucrose solution). The preference of 30 individuals was recorded for each type of bullseye size combination (small versus medium, and medium versus large). Among this, 15 bumblebees were presented the first type on the right, and the 15 others on the left. Statistical differences were calculated using a one-sample *t* test [RStudio (Version 1.1.1717)]. The second preference test was subsequently performed to identify consistency in preference over 10 choices in a larger foraging display. Ten rewarding discs, five of each bullseye size, were presented simultaneously, and the 10 first choices made by an individual forager were recorded. Statistical analyses were performed using RStudio (Version 1.4.1717), using a two-sided *t* test, assessing significant increase in % of one disc type chosen from than would be expected at random (50%).

#### Foraging speed experiments

Three artificial flowers, all from the same type (bullseye size from type 1), and offering 30 μl of 30% sucrose solution, were set 30 cm apart from one another in the arena (in position 1). A naïve individual was introduced to the arena and the times it took to move between the three discs (disc 1 to disc 2, then disc 2 to disc 3) during a foraging bout were recorded with a Samsung Galaxy Tab E tablet. A large reward (100 μl of 30% sucrose solution) was offered to the bee at the end of the foraging bout to encourage it to return to the hive. Flowers with which the bees came into contact were cleaned with 70% ethanol, and then water to remove scent marks. Then, the same bumblebee was offered to repeat the same experiment with the second type of artificial flowers (bullseye size, type 2), set 30 cm apart from one another in a new location (position 2). The same experiment, with the same bee, was repeated with the third disc type (type 3) at a new position in the arena (position 3). The entire procedure was then repeated at least five times to ensure that five complete foraging bouts on each flower type were recorded for each individual bee (15 foraging bouts total for each bee). This routine allowed us to control the variability in foraging speed between foragers (as each bee performed the experiment on each type of flower) and any potential effect of the position of the flowers in the arena. In total, 15 individuals were independently tested. The time taken for each bee to travel between each disc was extracted from the recordings using VLC Video Software and the Time v3.2 extension. After examining the plots of residuals, a single-factor analysis of variance (ANOVA) and a post hoc Tukey’s test were conducted in RStudio (Version 1.4.1717) to explore differences between artificial flower types.
